# Biopharmaceutical Understanding of Excipient Variability on Drug Apparent Solubility Based on Drug Physicochemical Properties. Case Study: Superdisintegrants

**DOI:** 10.1208/s12248-019-0406-y

**Published:** 2020-02-11

**Authors:** Panagiota Zarmpi, Talia Flanagan, Elizabeth Meehan, James Mann, Nikoletta Fotaki

**Affiliations:** 1 Department of Pharmacy and Pharmacology, University of Bath, Bath, BA2 7AY UK; 2 Pharmaceutical Technology & Development, AstraZeneca, Macclesfield, UK; 3 Present Address: UCB Pharma, Chemin du Foriest, B-1420 Braine-l’Alleud, Belgium

**Keywords:** croscarmellose sodium, crospovidone, drug solubility, excipient variability, sodium starch glycolate

## Abstract

The presence of different excipient types/brands in solid oral dosage forms may affect product performance and drug bioavailability. Understanding the biopharmaceutical implications of superdisintegrant variability (changes in material properties), variation (changes in excipient amount) and interchangeability (use of different excipient types with the same intended functionality) in oral drug performance would be beneficial for the development of robust final dosage forms. The current study investigated the impact of superdisintegrants (sodium starch glycolate, croscarmellose sodium, crospovidone) on the apparent solubility of drugs with different physicochemical properties (drug ionisation, drug lipophilicity, drug aqueous solubility). Compendial and biorelevant media were used to assess the impact of gastrointestinal conditions on the effects of excipient on drug apparent solubility. For the majority of compounds, changes in drug apparent solubility were not observed in superdisintegrant presence, apart from the cases of highly ionised compounds (significant decrease in drug solubility) and/or compounds that aggregate/precipitate in solution (significant increase in drug solubility). Excipient variability did not greatly affect the impact of excipients on drug apparent solubility. The use of multivariate data analysis identified the biopharmaceutical factors affecting excipient performance. The construction of roadmaps revealed that superdisintegrants may be of low risk for the impact of excipients on oral drug performance based on drug solubility alone; superdisintegrants activity could still be a risk for oral bioavailability due to their effects on tablet disintegration.

**Electronic supplementary material:**

The online version of this article (10.1208/s12248-019-0406-y) contains supplementary material, which is available to authorized users.

## INTRODUCTION

Introduction of the Quality by Design (QbD) initiative in pharmaceutical development requires the scientific understanding of the components and processes affecting final product qualities (1). The critical role of excipients in product performance and oral bioavailability is highlighted as presence of excipients in oral formulations may affect the biopharmaceutical profile of drugs with potential implications on drug absorption (2,3). Excipient variability or variation and the use of different excipients with the same intended functionality may further complicate the impact of excipients on oral drug bioavailability (4). The heterogeneous composition in the different regions of the gastrointestinal tract may as well modify the properties and functionality of excipients and presents an additional challenge to assess the impact of excipients on product performance (4).

Superdisintegrants are commonly used in immediate release formulations as they promote fast tablet disintegration and improve drug dissolution. Sodium starch glycolate (SSG), croscarmellose sodium (CCS) and crospovidone (CPV) are three commonly used crosslinked superdisintegrants due to their ability to adsorb water and/or swell in low concentrations (typically 2–8% for SSG (5), 0.5–5% for CCS (6) and 2–5% *w*/*w* for CPV (7) in tablet formulations (8)). SSG (Supplementary Fig. 1a) and CCS (Supplementary Fig. 1b) are sodium salts and their ionisation state differs between acidic (neutral form) and basic (ionised form) conditions, while CPV is a non-ionic polymer (Supplementary Fig. 1c). Swelling and shape recovery are the main suggested mechanisms by which superdisintegrants induce tablet disintegration. Swelling refers to the volumetric expansion of excipient particles due to water adsorption while shape recovery refers to excipient deformation upon contact with water (9). Real-time magnetic resonance imaging identified that SSG and CCS act through swelling while CPV acts through shape recovery (10). The limited knowledge on superdisintegrant molecular structure, interplay with other pharmaceutical components and performance in the gastrointestinal conditions is challenging for manufacturers (9). Superdisintegrant interchangeability could be questioned without appropriate identification of the biopharmaceutical consequences of their use.

Molecular properties (composition), particle properties (specific surface area, particle size distribution (PSD)) and level have been identified as the critical material attributes affecting excipient performance (for CPV, molecular properties were not critical) (4). For SSG and CCS, the degree of substitution and degree of crosslinking are critical functional properties. The degree of substitution (presence of the carboxymethyl group) increases polymer hydrophilicity and swelling (11). The degree of crosslinking reduces the excipient soluble content which can increase the viscosity of the surrounding medium and compromise tablet disintegration (4). PSD affects the swelling capacity of SSG and CCS, as larger particles swell more extensively compared to smaller particles (11). For CPV which exhibits a more porous structure, PSD relates to water uptake as the higher porosity of larger particles results in faster water adsorption and tablet disintegration (12). Finally, increasing excipient level in tablet formulations leads to faster water uptake and tablet disintegration, but care should be taken when using gelling superdisintegrants as high excipient levels may result in the formation of viscous layers around drug particles (13).

Beyond the intended superdisintegrant use (facilitation of dosage form disintegration), the biopharmaceutical implications of superdisintegrants on drug solubility, drug permeability or drug–excipient interactions are not fully understood. The pH of the medium affects the performance of SSG and CCS due to the ionisation pattern of the excipients. The swelling ability of the neutral form is reduced due to its low hydration capacity compared to the ionised form (8). The performance of superdisintegrants can also relate to drug physicochemical properties. Electrostatic interactions between cationic drugs and the carboxyl group of SSG and CCS are known to affect the percentage of drug recovery during routine drug analysis (14,15) or delay drug release from tablet formulations (16). Drug–excipient interactions are affected by the presence of salts, as high salt concentrations suppress the binding of drugs in the hydrogels (17). Adsorption of lipophilic molecules to CPV through hydrophobic interactions has been reported (17) that could also affect drug release from pharmaceutical formulations.

The aim of this study was to investigate the biopharmaceutical implications and criticality of superdisintegrant variability and variation on drug apparent solubility. The impact of excipient variability on drug apparent solubility was studied by selecting three SSG brands of different viscosity type and two CCS and CPV brands of different PSD. Two excipient levels (low, 2% *w*/*w*; high, 5% *w*/*w*) were used to assess the impact of excipient variation on drug apparent solubility. The biopharmaceutical implications of superdisintegrant variability were evaluated by choosing compounds with different physicochemical properties (drug ionisation, drug lipophilicity, drug aqueous solubility) and media (compendial and biorelevant) representing the gastric and intestinal compartments. The significance of drug properties, excipient presence and medium characteristics on the effects of superdisintegrants on drug apparent solubility were investigated with the use of multivariate data analysis (partial least squares (PLS)) and the design of roadmaps.

## MATERIALS AND METHODS

### Materials

APIs: Sulfamethoxazole (SMX) and paracetamol (PRC) were obtained from Fisher Scientific (UK). Furosemide (FRS), itraconazole (ITZ) and dipyridamole (DPL) were obtained from VWR (UK). Ibuprofen (IBU), carbamazepine (CBZ) and metformin (MTF) were obtained from Fagron (UK). Excipients: Glycolys LV and Glycolys (Roquette, France), Explotab CLV (JRS Pharma, USA), Kollidon CL-F and Kollidon CL (BASF-SE, Germany), AcDiSol (FMC, USA) and Primellose (DFE Pharma, Germany) were obtained from the specified sources. Chemicals: Acetic acid (>99.7%), hydrochloric acid 36.5–38%, high-performance liquid chromatography (HPLC)-grade methanol, HPLC-grade acetonitrile, dichloromethane and pepsin (from porcine) were obtained from Sigma-Aldrich (UK). Maleic acid, sodium chloride, sodium hydroxide, potassium phosphate monobasic, sodium dihydrogen orthophosphate dihydrate, disodium hydrogen orthophosphate dihydrate, potassium dihydrogen orthophosphate, anhydrous sodium sulfate and HPLC-grade trifluoroacetic acid were obtained from Fisher Scientific (UK). Sodium taurocholate (Prodotti Chimici Alimentari S.P.A., Italy) and egg lecithin–Lipoid EPCS (Lipoid GmbH, Germany) were obtained from the sources specified. Water was ultra-pure (Milli-Q) laboratory grade. Filters: Whatman® 13 mm cellulose nitrate filters 0.45 μm pore size and polytetrafluoroethylene (PTFE) 13 mm filter 0.45 μm pore size were purchased from Fisher Scientific (UK).

### Instrumentation

Fisherbrand waterbath (Fisher Scientific, UK), Sartorius BP 210 D balance (Sartorius Ltd., UK), Buchi R114 Rotavapor (Buchi, Switzerland), Mettler Toledo SevenCompact S210 pH meter (Mettler Toledo, Switzerland), Vortex-Genie 2 vortex mixer (Scientific Industries Inc., USA), Brookfield HA-RVIII viscometer (Brookfield Ametek, USA), Agilent Technologies 1100 series HPLC system (quaternary pump (G1311A), autosampler (G1313A), thermostated column compartment (G1316A), diode array detector (G1329A)) and Chemstation software (Agilent Technologies, USA).

### Methods

#### Compounds Selected for Solubility Experiments

The choice of the compounds for the solubility experiments was based on the biopharmaceutical properties affecting drug solubility, dissolution and permeability through the gastrointestinal tract (18). The compounds covered a range of properties in terms of ionisation (low ionised—*F*_(ion)_ < 50%, highly ionised—*F*_(ion)_ > 50%), lipophilicity (based on the drugs’ partition coefficient (log *P*), −1.5 < log *P* < 6.5) and aqueous solubility (based on the compound’s BCS (Biopharmaceutical Classification System) classification (high—BCS class I and III; low—BCS class II and IV)) (19). The compounds used for the solubility experiments, their physicochemical properties (drug ionisation, drug lipophilicity, drug aqueous solubility) and their structure are presented in Table I.

**Table I:**
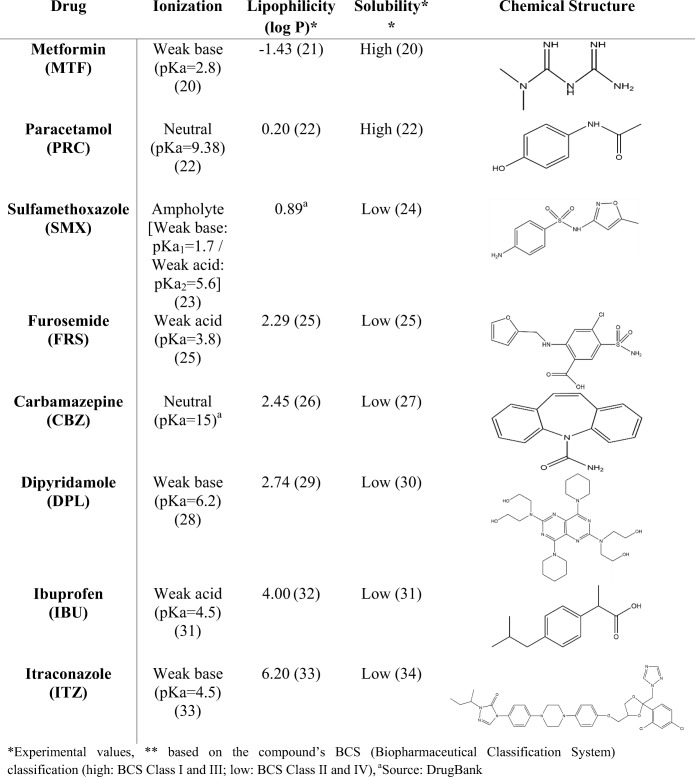
Physicochemical Properties and Structure of the Compounds Used for the Solubility Experiments (ChemDraw Professional 15)

#### Media Prepared for Solubility Experiments

Compendial media (0.1 N HCl pH 1, phosphate buffer pH 6.8) were prepared according to the method described in European Pharmacopoeia (35). Fasted State Simulated Gastric Fluid (FaSSGF) and Fasted State Simulated Intestinal Fluid (FaSSIF-V2) were prepared as described by Jantratid *et al*.(36).

#### Design of Experiments (DoE) Used for Solubility Experiments

The number of experiments was determined with a full-factorial Design of Experiments (DoE) using StatGraphics Centurion XVII (Statpoint Technologies Inc., USA). As changes in drug solubility are expected according to the composition of the studied media (pH, presence of bile salts), two models for the DoE were constructed to discriminate between the effects of excipients on drug apparent solubility in compendial (model 1) and biorelevant conditions (model 2). The examined factors were (1) compound (Table I), (2) excipient brand (SSG—Glycolys LV, Explotab CLV, Glycolys; CCS—AcDiSol, Primellose; CPV—Kollidon CL-F, Kollidon CL), (2) excipient level (low, high) and (4) medium (gastric, intestinal). The impact of each excipient on drug apparent solubility (expressed as the relative increase or decrease in presence compared to absence of excipient (‘Treatment of *In Vitro* Solubility Data’ section)) was set as the response. A total of 224 × 3 experiments were determined for each model. In addition, 16 × 3 additional experiments in triplicate for each model were conducted to determine drug solubility in the corresponding media in the absence of excipient. These experiments were not included in the DoE as drug solubility in excipient absence was measured only for the calculation of relative excipient effects on drug solubility.

### Characterisation of Superdisintegrants

#### Viscosity Measurements

Samples of each superdisintegrant were prepared as 3% *w*/*v* dispersions in water. Then 0.5 mL of each sample was loaded into the cup of a rotational viscometer. The viscosity of each dispersion was measured every 10 min for an hour at 25°C using a CPA-40z spindle rotated at a speed of 120 rpm (37). All experiments were performed in triplicate.

#### Particle Size Distribution

The PSD of the studied CCS and CPV brands was measured using laser diffraction (dry dispersion) and the cumulative undersized particle parameters *d*_10_ (μm), *d*_50_ (μm) and *d*_90_ (μm) were calculated (data kindly provided by AstraZeneca).

#### Solubility Studies

Drug solubility studies in the absence and presence of excipient were performed in triplicate using the shake-flask method (38). Drug excess amount and 2% *w*/*w* or 5% *w*/*w* of each excipient were weighed and placed in centrifuge tubes. For poorly soluble drugs, the amount of excipient was determined considering an average of 500 mg tablet weight (39) which resulted in 9% *w*/*w* (10 mg of excipient and 100 mg of drug; low level) and 20% *w*/*w* (25 mg of excipient and 100 mg of drug; high level) of excipient in the total volume of the physical mixture. For highly soluble drugs, as higher drug excess amount was used to ensure saturation, the excipient amount was increased in order to keep the same percentage *w*/*w* of excipient in the total volume of the physical mixture as per the poorly soluble drugs. The physical mixtures were vortexed for 3 min. Then 5 mL of each medium was added in the tubes and the samples were placed in a shaking water bath (37°C, 200 strokes per minute (spm)). At 0.5, 4 and 24 h (for PRC, SMX, CBZ, DPL, IBU) and at 24 h (for MTF, FRS, ITZ), 500 μL was sampled and filtered through PTFE filters (or cellulose nitrate filters for the cases of IBU and CBZ). Filter adsorption studies were prior performed in triplicate for each drug. No adsorption issues onto the filters used were observed for the studied drugs. Filtered samples were further diluted (if needed) with the corresponding medium and analysed by HPLC (Supplementary Table I). Analytical HPLC procedures for drug quantification in the samples were modifications of already published methods. Drug quantification was made based on calibration curves. Standards were formulated from concentrated stock solution consisting of drug dissolved in MeOH. The pH of samples after the completion of each experiment was measured to determine whether there is a change in the pH of the solution by the presence of dissolved drug (30) (that could result in a change in drug solubility at 24 h). Drug solubility was calculated based on the sample drug concentration measured. Solubility values measured experimentally for neutral drugs, for weak acids in acidic media and for weak bases in basic media determined the intrinsic solubility values. Solubility values measured experimentally in basic media (for weak acids) and acidic media (for weak bases) determined drug solubility of the ionised molecules. The drug solubility measured was considered as the apparent drug solubility (dynamic solubility), as experimental points over a period of time were not available for the whole set of drugs to ensure that equilibrium solubility has been reached in 24 h for all the studied compounds.

#### Treatment of *In Vitro* Solubility Data

The relative effect (RE) of each excipient on drug apparent solubility was calculated based on Eq. 1:

1 $$ RE=\frac{\left(S- Sr\right)}{Sr}\times 100 $$

where *S* and *Sr* denote drug solubility in presence and absence (reference solubility) of excipient at 0.5, 4 and 24 h. REs of excipients on drug solubility >25% or <−20% were considered as significant change in drug apparent solubility to assess excipient criticality (this range was selected as a similar range is set in order to assess differences in drug exposure after oral administration; *i.e.* in bioequivalence studies) (40).

Box plots depicting the impact of excipients on drug solubility at 24 h for all the studied compounds or as a function of time (0.5, 4 and 24 h) for CBZ were constructed using Spotfire 7.10.1 (TIBCO software Inc., USA). The classification gradient maps portraying the impact of the studied brands on drug solubility at 24 h as a function of drug aqueous solubility were generated using SigmaPlot 13.0 (Systat Software Inc., USA).

In cases where drug intrinsic solubility was not determined experimentally (SMX and DPL in compendial and biorelevant media), the theoretical intrinsic solubility was calculated using the solubility–pH equations (Eqs. 2–5) (41):

2 $$ \log S=\log {S}_o+\log \left({10}^{- pKa+ pH}+1\right)\ \mathrm{for}\ \mathrm{weak}\ \mathrm{acids} $$

3 $$ \log S=\log {S}_o+\log \left({10}^{pKa- pH}+1\right)\ \mathrm{for}\ \mathrm{weak}\ \mathrm{bases} $$

4 $$ \log S=\log {S}_o+\log \left({10}^{+p{Ka}_2+{pKa}_1-2 pH}+{10}^{pKa_2- pH}+1\right)\ \mathrm{for}\ \mathrm{diprotic}\ \mathrm{bases} $$

5 $$ \log S=\log {S}_o+\log \left({10}^{+{pKa}_1- pH}+{10}^{-{pKa}_2+ pH}+1\right)\mathrm{for}\ \mathrm{ampholytes} $$

where *S* and *S*_*o*_ indicate drug solubility at the given pH and the intrinsic solubility, respectively. These equations provide a simplified view for the determination of drug solubility values as deviations from these models (in cases of drug aggregation or drug solubilisation in the biorelevant media) can be anticipated (41). The theoretical intrinsic solubility values were calculated based on the final pH and the experimental solubility values of the ionised weak acids (in basic media) and weak bases (in acidic media). Theoretical pH–solubility profiles in the physiological pH range were constructed to assess if changes in the pH of the medium could justify differences in drug solubility by excipient presence. The final pH and intrinsic solubility values (experimental or theoretical) were used for the construction of the theoretical pH–solubility profiles in the physiological pH range based on Eqs. 2–5.

#### Multivariate Data Analysis

Excipient REs on drug apparent solubility were correlated to drug physicochemical properties (drug ionisation, drug lipophilicity, drug aqueous solubility), excipient critical material attributes (viscosity for SSG, PSD for CCS and CPV, level) and medium characteristics (gastric, intestinal) by partial least squares (PLS) regression using the XLSTAT software (Microsoft, USA). Two models for the REs of excipients on drug apparent solubility in compendial media (model 1) and biorelevant media (model 2) were constructed. The evaluated variables for both models were categorised according to their type as categorical (expressing a category or type) and numerical (measurements with numerical meaning). Categorical variables included (1) drug solubility (low, high), (2) amine group (absence, presence), (3) excipient brand (low and high PSD for CCS and CPV), (4) excipient level (low, high) and (5) medium (gastric, intestinal), while numerical parameters included (1) theoretical percentage of drug ionised (*F*_ion_; calculated based on the Henderson–Hasselbalch equation at the pH of each medium), (2) drug lipophilicity (log *P*) and (3) excipient brand (viscosity in cP of dispersion after 1 h for SSG). Excipient REs on drug solubility at 24 h were used as the response. The selected interaction terms included each excipient property combined with each drug physicochemical property (drug ionisation, drug lipophilicity, drug aqueous solubility) and medium characteristics (gastric, intestinal). Observation diagnostics were performed prior to model analysis to identify outliers in the data set. The distance of each observation to the model in the *Y*-plane (DmodY) tool based on PLS residuals was used. Plots of standardised DmodY *versus* each observation were generated and any observation exceeding the maximum tolerance volume in *Y* (Dcrit_(*Y*)_) was considered an outlier (42,43). Exclusion of outliers was based on two criteria: (1) deviating cases (positive REs) in solubility caused by a pH shift of the solution; (2) observations resulting in high variability (coefficient of variation (CV%) > 20%) within the triplicate samples (one value from the triplicate could be excluded as the outlier analysis could detect these values). PLS models generated with and without outlier exclusion (data not shown) confirmed that outlier exclusion did not alter the interpretation of results but only enhanced the predictive ability of the regression model. The generated models were assessed in terms of goodness of fit (*R*^2^) and goodness of prediction (*Q*^2^). High values of *R*^2^ and *Q*^2^ with a difference not greater than 0.2–0.3 were indications of successful models (44). The number of PLS components (lines on the *X*-space which best approximate and correlate with the *Y*-vector) was based on minimum predictive residual sum of squares (PRESS) (44). From the available components, the one at which *Q*^2^ reached its maximum value was selected (42). Standardised coefficients were used to show the direction (positive or negative) and extent of each variable on the response. The significance of the selected variables was assessed by the variable influence on projection (VIP) value. VIP values >0.8 were considered as moderately influential in the model while VIP values >1 were considered the most influential in the model (44). A 95% confidence interval was used.

#### Roadmap Design

The risks of superdisintegrant variability on drug apparent solubility in a biopharmaceutical perspective was demonstrated with the use of roadmaps by combining the impact of excipients on drug solubility at 24 h from the solubility studies to excipient (viscosity for SSG, PSD for CCS and CPV) and drug (drug ionisation, drug lipophilicity, drug aqueous solubility) physicochemical properties. Drugs were categorised according to drug aqueous solubility and drug lipophilicity (Table I) and drug ionisation (low ionised, *F*_(ion)_ < 50%; highly ionised, *F*_(ion)_ > 50%). The risk assessment of the impact of excipients on drug solubility was evaluated by setting reference range criteria of −20% to 25% (40) on the REs of excipient on drug solubility. REs of excipients on drug apparent solubility outside these values (REs < −20% or REs > 25%) were considered to be potentially significant for oral drug performance.

## RESULTS AND DISCUSSION

### Characterisation of Superdisintegrants

The viscosity data of the studied excipient types and brands are presented in Table II. The viscosity of a superdisintegrant dispersion with time relates to the degree of crosslinking (37). The SSG dispersions exhibit higher viscosity values compared to the CCS or CPV dispersions indicating that the SSG brands contain higher soluble material content compared to the CCS and CPV brands, which increases the viscosity of the dispersion over time (9). The higher degree of crosslinking for Glycolys LV and Explotab CLV explains the lower viscosity of their aqueous dispersion compared to the dispersion of Glycolys, as fewer polymeric chains are able to dissolve in the surrounding medium. Differences in the viscosity of dispersions between the different brands of CCS and CPV are not revealed. Experimental data of PSD (kindly provided by AstraZeneca) for the studied CCS and CPV brands are summarised in Table II. AcDiSol comprised smaller particles compared to Primellose; therefore, the CCS brands will be referred as CCS(L) (AcDiSol) and CCS(H) (Primellose) in the sections below. Differences in the PSD were also observed for the CPV brands, as the particle size of Kollidon CL-F was smaller compared to Kollidon-CL and therefore Kollidon CL-F and Kollidon-CL will be referred as CPV(L) and CPV(H), respectively, in the sections below.

**Table II:** (I) Viscosity (cP) of the Studied Superdisintegrant Brands (Mean ± SD) and (II) Particle Size Distribution of the Studied CCS and CPV Brands

I. Viscosity values							
Time (min)	SSG		CCS			CPV	
	Glycolys LV	Explotab CLV	Glycolys	CCS(L)	CCS(H)	CPV(L)	CPV(H)
10	9.7 (± 0.3)	11.7 (± 0.6)	18.6 (± 1.6)	8.2 (± 1.1)	9.1 (± 0.7)	1.6 (± 0.1)	2.4 (± 0.2)
20	9.9 (± 0.3)	11.9 (± 0.6)	19.1 (± 1.8)	7.6 (± 0.8)	8.8 (± 0.8)	1.5 (± 0.1)	2.0 (± 0.3)
30	10.1 (± 0.3)	12.1 (± 0.6)	19.6 (± 2.0)	7.5 (± 0.7)	8.4 (± 0.7)	1.5 (± 0.1)	1.9 (± 0.3)
40	10.3 (± 0.3)	12.3 (± 0.5)	19.9 (± 2.1)	7.5 (± 0.7)	8.0 (± 0.9)	1.5 (± 0.1)	1.7 (± 0.3)
50	10.5 (± 0.3)	12.4 (± 0.24)	20.3 (± 2.2)	7.5 (± 0.6)	8.2 (± 0.9)	1.5 (± 0.1)	1.6 (± 0.1)
60	10.6 (± 0.2)	12.7 (± 0.3)	20.6 (± 2.3)	7.5 (± 0.5)	7.6 (± 0.8)	1.5 (± 0.0)	1.6 (± 0.2)
II. Particle size distribution							
	CCS			CPV			
	CCS(L)		CCS(H)	CPV(L)		CPV(H)	
*d*_10_ (μm)	12.8		21.8	12.1		15.9	
*d*_50_ (μm)	31.9		52.2	36.3		77.6	
*d*_90_ (μm)	74.2		109.8	117.4		234.3	

### Solubility Studies

#### Impact of Superdisintegrants on Drug Apparent Solubility

The reference drug solubility values in compendial and biorelevant media at 24 h are summarised in Table III. From the studied compounds, only weak acid or weak bases showed a pH-dependent solubility, as expected. For neutral drugs or weak acids/weak bases in media where drugs are unionised, reference solubility values were higher in biorelevant compared to compendial media due to the presence of solubilising components (45). For weak acids or weak bases (except from MTF) in media where drugs are highly ionised, the higher percentage of drug ionised resulted in increased reference drug solubilities in compendial (0.1 N HCl pH 1, phosphate buffer pH 6.8) compared to biorelevant media (FaSSGF pH 1.6, FaSSIF-V2 pH 6.5) (46).

**Table III:** Reference Solubility Values (μg/mL) of the Studied Drugs in Compendial and Biorelevant Media (Mean ± SD)

	Compendial media		Biorelevant media	
Drug	0.1 N HCl pH 1	Phosphate buffer pH 6.8	FaSSGF	FaSSIF-V2
MTF	3.1 × 10^5^ (± 0.3 × 10^5^)	3.1 × 10^5^ (±0.2 × 10^5^)	3.4 × 10^5^ (± 0.8 × 10^5^)	4.3 × 10^5^ (± 0.4 × 10^5^)
PRC	1.6 × 10^4^ (± 0.1 × 10^4^)	1.5 × 10^4^ (± 0.1 × 10^4^)	1.7 × 10^4^ (± 0.2 × 10^4^)	1.7 × 10^4^ (± 0.1 × 10^4^)
SMX	1.6 × 10^3^ (± 0.1 × 10^3^)	3.7 × 10^3^ (± 0.1 × 10^3^)	862 (± 21)	1.3 × 10^3^ (± 0.1 × 10^3^)
FRS	14 (± 2)	3.4 × 10^3^ (± 1.4 × 10^2^)	15 (± 1)	1.6 × 10^3^ (± 3.0 × 10^2^)
CBZ	265 (± 6)	227 (± 9)	368 (± 1)	280 (± 7)
DPL	1.3 × 10^4^ (± 9.1 × 10^2^)	5 (± 1)	8.6 × 10^3^ (± 2.0 × 10^2^)	13 (± 1)
IBU	43 (± 3)	5.5 × 10^3^ (± 6.7 × 10^2^)	44 (± 5)	1.5 × 10^3^ (± 5.8)
ITZ	11 (± 1)	–^*a*^	1.2 (± 0.2)	0.05 (± 0.01)

*MTF* metformin, *PRC* paracetamol, *SMX* sulfamethoxazole, *FRS* furosemide, *CBZ* carbamazepine, *DPL* dipyridamole, *IBU* ibuprofen, *ITZ* itraconazole

^*a*^Below limit of detection of the chromatographic method

**Fig. 1:**
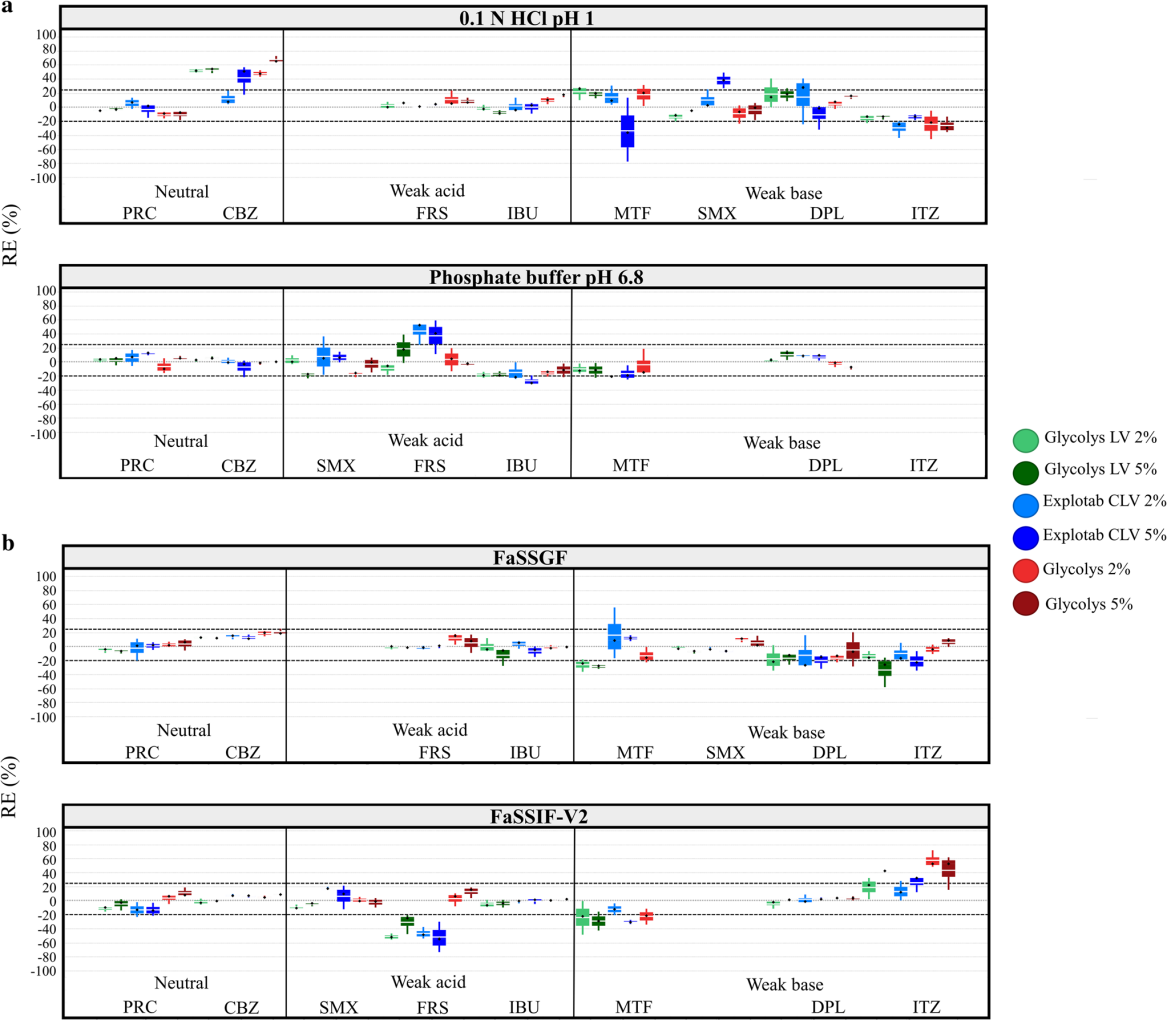
Box plots of the relative effects (%) of the studied SSG brands on drug solubility at 24 h in **a** compendial and **b** biorelevant media. The excipient brands are shown as Glycolys LV (green colour), Explotab CLV (blue colour) and Glycolys (red colour). Light and dark colours correspond to low and high excipient level, respectively (mean—white line, median—black diamond, *n* = 3)

**Fig. 2:**
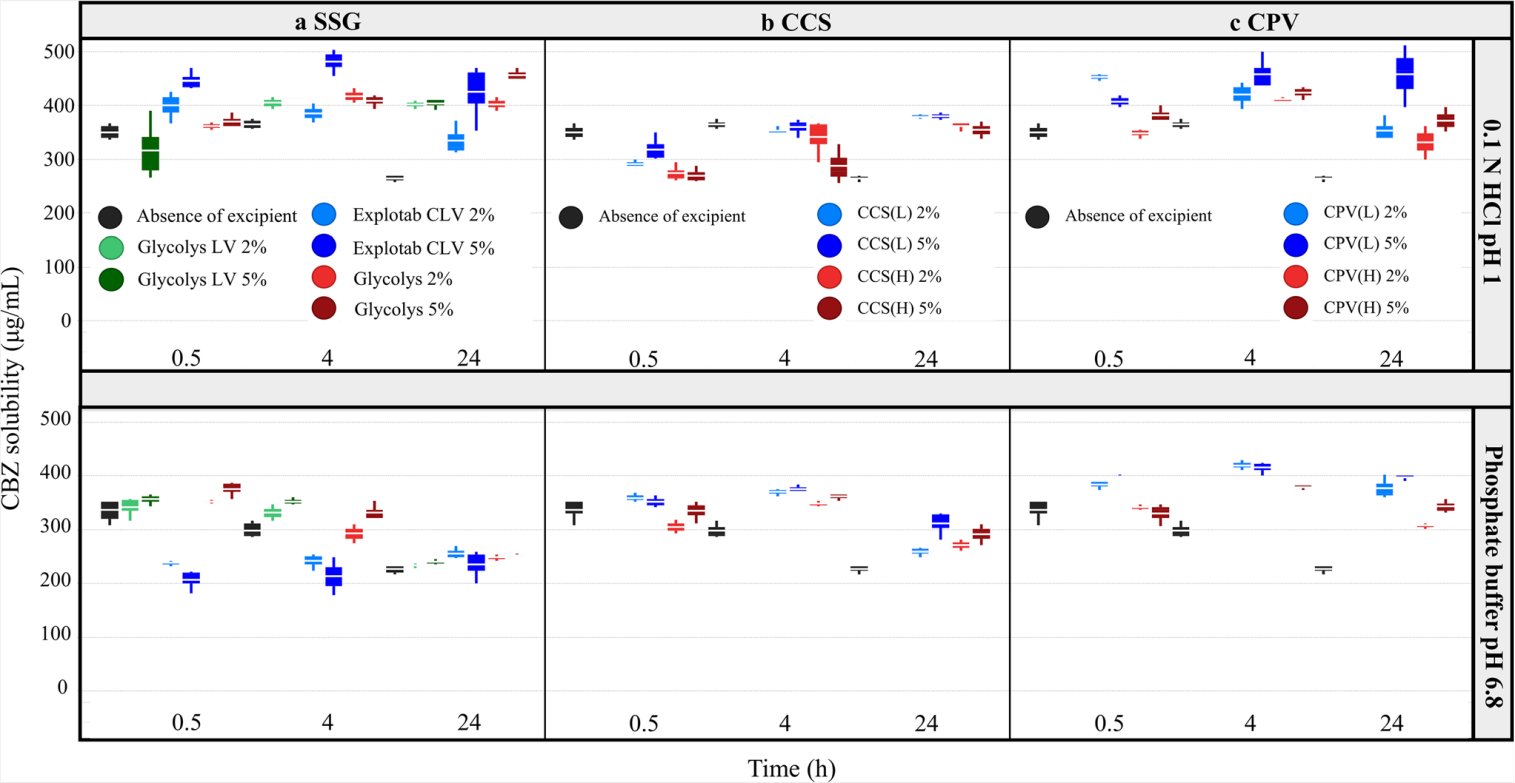
Box plots of CBZ solubility (μg/mL) in absence (black colour) and presence of the studied **a** SSG (Glycolys LV (green colour), Explotab CLV (blue colour), Glycolys (red colour)), **b** CCS (CCS(L) (blue colour), CCS(H) (red colour), CPV(L) (blue colour), CPV(H) (red colour)) brands in 0.1 N HCl pH 1 and phosphate buffer pH 6.8. Light and dark colours correspond to low and high excipient level, respectively (mean, *n* = 3)

*SSG*: The effects of the studied SSG brands on drug solubility at 24 h in compendial and biorelevant media are presented in Fig. 1, respectively. For MTF, in presence of 5% of Glycolys the solubility experiments resulted in the creation of a paste due to the high viscosity of the polymer in all media tested; therefore, only results with the low Glycolys level are presented. Significant reduction in drug apparent solubility by the low-viscosity SSG brands (Glycolys LV, Explotab CLV) was observed for weak acids and weak bases in media where drugs are highly ionised (−50% < REs < −20%). The high viscosity Glycolys significantly decreased the MTF solubility in FaSSIF-V2 (RE = −22%, low excipient level) and ITZ solubility in 0.1 N HCl pH 1 (REs of −23% and −25% for the low and high excipient level, respectively) at 24 h. Reduction in the pH of basic media for weak acids (0.2–0.7 pH units) was observed (attributed to the drug ionisation) in the cases where SSG significantly decreased drug solubility. Changes in the pH of the media cannot justify the differences in drug solubility for weak acids in excipient presence as experimental drug solubility values do not correspond to the theoretical equilibrium solubility values (expected by the change in the pH of the medium and the design of the pH–solubility profiles) (Supplementary Fig. 2). Increase in the pH of acidic media for MTF, DPL and ITZ (0.2–4 pH units) was observed in the cases where SSG presence significantly decreased drug solubility (attributed to drug ionisation) however the impact of pH on the solubility of the aforementioned weakly basic compounds cannot be assessed due to *in situ* salt formation between the API and counterions of the medium (47). Presence of insoluble excipients may delay drug dissolution and/or drug solubilisation as their insolubility or variable ‘wetting’ characteristics result in reduced drug–medium contact (48). Therefore, the observed reduction in apparent drug solubility by SSG could relate to a shielding excipient effect (polymer adsorption around drug particles) on powder surface which further retards the time at which drug equilibrium solubility in the medium is reached (polymer adsorption on drug particles would not affect the true drug equilibrium solubility). The adsorption of ionised polymers around drug particles could also induce changes in the local surface pH, as compared to the pH of the bulk solution, and affect the dissolution of weak acids or weak bases (49). Cases of decreased drug solubility at 24 h were mostly observed in presence of low-viscosity SSG brands (Glycolys LV, Explotab CLV) compared to the high-viscosity Glycolys and could be explained by the extensive swelling of low-viscosity brands (9) which creates a barrier for drug dissolution and/or drug solubilisation from the powder surface. For neutral drugs, significant increase in drug apparent solubility was observed for CBZ in 0.1 N HCl pH 1 (42% < REs < 67%) in presence of all the studied brands (Fig. 1). Changes in the pH of the media were not observed in the case of CBZ in excipient presence or absence. Solubility data of CBZ at 0.5, 4 and 24 h in absence and presence of the studied SSG brands in compendial and biorelevant media are presented in Fig. 2a. The solubility of pure CBZ decreased through time in compendial media (350 μg/mL and 250 μg/mL at 0.5 h and 24 h, respectively), potentially due to drug aggregation (50) or due to the conversion of CBZ anhydrate to CBZ dihydrate in solution (solution mediated phase transformation) (51,52). This reduction in CBZ apparent solubility is not observed in presence of SSG, as potentially dissolved polymer particles may enhance drug solubilisation and delay drug aggregation (53). Inhibition of the solution mediated phase transformation of CBZ in excipient presence due to the interaction of the amine group of CBZ with the carboxylic group of SSG (Supplementary Fig. 1) could also explain the fact that CBZ apparent solubility was not reduced in excipient presence (51,52) and justify the less pronounced impact of SSG in phosphate buffer pH 6.8 compared to 0.1 N HCl pH 1 (as the increased excipient hydrophilicity (due to excipient ionisation (8)) would decrease the likelihood of drug–excipient interaction (54)). For weak acids, significant increase in drug solubility at 24 h was observed in presence of Explotab CLV for FRS in phosphate buffer pH 6.8 (REs of 44% and 37% for the low and high level, respectively) (Fig. 1). In this case, the reduction in the pH of the medium was higher in presence (FRS—0.3 pH units) compared to excipient absence (FRS—0.2 pH units). Evaluation of the theoretical pH–solubility profile (Supplementary Fig. 2) revealed that in SSG presence, the experimental drug solubility corresponds to the theoretical equilibrium solubility (expected by the change in the pH of the medium); therefore, the aforementioned case of increased solubility is attributed to the shift in the pH of the medium (further investigations on the impact of dissolved drugs or excipients on the pH of the medium are needed to explain the nature of this change, as reduction in the pH of the medium by SSG is not expected). For weak bases, significant increase in drug solubility at 24 h was observed for SMX in 0.1 N HCl pH 1 in presence of Explotab CLV (high excipient level—RE = 38%) (Fig. 1). The observed differences in the pH of the medium in excipient presence (−0.2 pH units) compared to excipient absence (−0.06 pH units) explain the differences in drug solubility as theoretical drug solubility in presence of Explotab CLV corresponds to the theoretical equilibrium value (Supplementary Fig. 2). Increase in drug apparent solubility was also observed for ITZ in FaSSIF-V2 in presence of the high excipient level of low viscosity brands (REs of 42% and 25% for Glycolys LV and Explotab CLV, respectively) and both levels of the high-viscosity Glycolys (REs of approximately 50% for both excipient levels) (Fig. 1). Changes in the pH of the medium in presence of SSG were not observed in this case despite the ionisation pattern of the excipient potentially due to the buffer capacity of the medium (10 mM/dpH) (45). ITZ forms a supersaturated solution in FaSSIF-V2 due to the micellar solubilisation effect of bile salts and slowly precipitates with time (55). The increase in ITZ apparent solubility in SSG presence in FaSSIF-V2 can be attributed to the inhibition of drug precipitation by the polymeric chains of SSG. The increase in ITZ solubility at 24 h was more pronounced in presence of the high- compared to the low-viscosity brands, as potentially high-viscosity excipients have a better ability in delaying particle agglomeration and improve drug solubilisation (56).

**Fig. 3:**
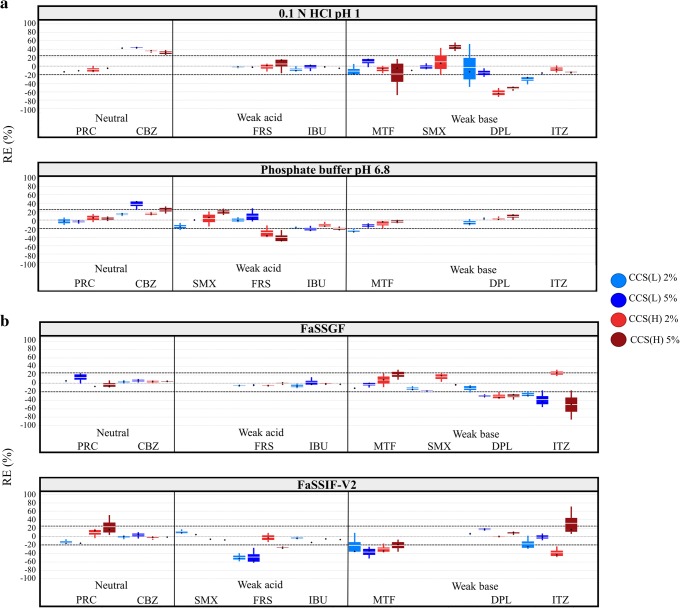
Box plots of the relative effects (%) of the studied CCS brands on drug solubility at 24 h in **a** compendial and **b** biorelevant media. The excipient brands are shown as CCS(L) (blue colour) and CCS(H) (red colour). Light and dark colours correspond to low and high excipient level, respectively (mean—white line, median—black diamond, *n* = 3)

*CCS*: Cases of significant decrease in the solubility of weak acids and weak bases in presence of CCS were mostly observed in media where drugs are highly ionised (CCS(L), −50% < REs < −20%; CCS(H), −62% < REs < −20%) (Fig. 3). Reduction in drug solubility at 24 h was also observed for ITZ in FaSSIF-V2 by the low level of CCS(H) (RE = −40%) (Fig. 3). In the cases of significant decrease in drug apparent solubility by CCS, changes in the pH of the media (0.2–0.7 pH unit reduction in basic media for weak acids, 0.2–4 pH unit increase in acidic media for MTF, DPL and ITZ) are attributed to drug ionisation and cannot explain the differences in drug solubility in presence of CCS (for weak acids) or be evaluated (for MTF, DPL and ITZ), as explained previously in the case of SSG (Supplementary Fig. 3). The slow drug dissolution and/or drug solubilisation by the presence of CCS particles on the surface of the powder could justify the pronounced decrease in drug apparent solubility by CCS (48). Significant increase in drug solubility at 24 h for neutral drugs was observed in the case of CBZ in 0.1 N HCl pH 1 (32% < REs < 43% for both levels of CCS(L) and CCS(H)) and in phosphate buffer pH 6.8 (RE = 37% for the high level of CCS(L)) (Fig. 3). As changes in the pH of the media in excipient presence were not observed for CBZ, the differences in CBZ apparent solubility in presence and absence of CCS are attributed to the enhanced drug solubilisation or inhibition of drug solution-mediated phase transformation by the excipient (54) (Fig. 2b). For weak acids, significant increase in drug solubility at 24 h in CCS presence was not observed. For weak bases, significant increase in SMX solubility at 24 h was observed in 0.1 N HCl pH 1 in presence of 5% CCS(H) (RE = 45%) and is attributed to the change in the pH of the medium, as the reduction in the pH of the medium was higher in presence (0.3 pH units) compared to absence of 5% CCS(H) (0.06 pH units) and the experimental and theoretical drug solubility in excipient presence are similar (Supplementary Fig. 3) (further investigations on the impact of dissolved drug or excipient are needed to explain the nature of this change, as reduction in the pH of the medium by CCS is not expected). For weak bases, significant increase in drug apparent solubility was observed for ITZ in FaSSIF-V2 in presence of 5% CCS(H) (RE = 31%) which could be justified by the enhanced drug solubilisation by the excipient.

**Fig. 4:**
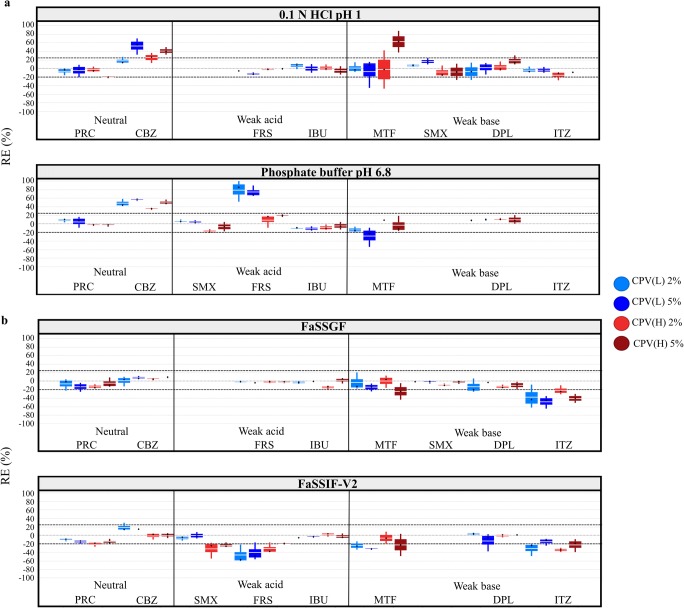
Box plots of the relative effects (%) of the studied CPV brands on drug solubility at 24 h in **a** compendial and **b** biorelevant media. The excipient brands are shown as CPV(L) (blue colour) and CPV(H) (red colour). Light and dark colours correspond to low and high excipient level, respectively (mean—white line, median—black diamond, *n* = 3)

*CPV*: Cases of significant reduction in drug apparent solubility by CPV presence was observed for weak acids and weak bases in media where drugs are highly ionised (CPV(L), −50% < REs < −20%; CPV(H), −40% < REs < −21%) (Fig. 4). Reduction in drug solubility at 24 h was also observed in the case of ITZ in FaSSIF-V2 in presence of both CPV brands (−30% < REs < −20%) (Fig. 4). In the case of significant reduction in drug apparent solubility by CPV, the ionisation of drugs resulted in reduction in the pH of the basic media for weak acids (0.2–0.7 pH units) or increase in the pH of acidic media for MTF, DPL and ITZ (0.2–4 pH units). The observed changes in the pH of the media cannot explain the differences in drug solubility in CPV presence (Supplementary Fig. 4), as explained previously for SSG and CCS. Therefore, the pronounced reduction in drug apparent solubility by CPV could relate to the presence of the insoluble excipient on the powder surface (48). For neutral drugs, significant increase in drug apparent solubility was observed in the case of CBZ in compendial media (25% < REs < 56%) and is attributed to the enhanced drug solubilisation or inhibition of drug solution mediated phase transformation by the excipient (Fig. 2c) (54). For weak acids, significant increase in drug solubility at 24 h was observed for FRS in phosphate buffer pH 6.8 in presence of both levels of CPV(L) (REs ≈ 70%) (Fig. 4). This pronounced increase in FRS apparent solubility is justified by the change in the pH of the medium, as the reduction in the pH of the medium was higher in excipient presence (0.4 pH units) compared to excipient absence (0.2 pH units) (Supplementary Fig. 4) (further investigations are needed to explain the nature of this change, as changes in the pH of the medium by the non-ionic CPV are not expected). For weak bases, significant increase in the 24-h solubility of MTF was observed in 0.1 N HCl pH 1 in presence of the high level of CPV(H) (RE = 63%) (Fig. 4). The increase in the pH of the medium in absence and presence of excipient was similar (3 pH units) and is attributed to the protonation of MTF. As changes in the pH of the media for weak bases cannot be evaluated, further investigations are needed to explain the pronounced increase in MTF apparent solubility.

The solubility data showed increased variability in the cases where superdisintegrant presence significantly affected drug solubility (MTF—CV% > 30%; PRC or highly ionised poorly soluble drugs—20% < CV% < 40%). As working with physical mixtures may yield high standard deviations due to the heterogeneous dispersion of the constituents (57,58), the increased variability can be attributed to the heterogeneous saturation of powder surface with excipient particles.

#### Impact of Excipients on Drug Apparent Solubility Based on Drug Physicochemical Properties

**Fig. 5:**
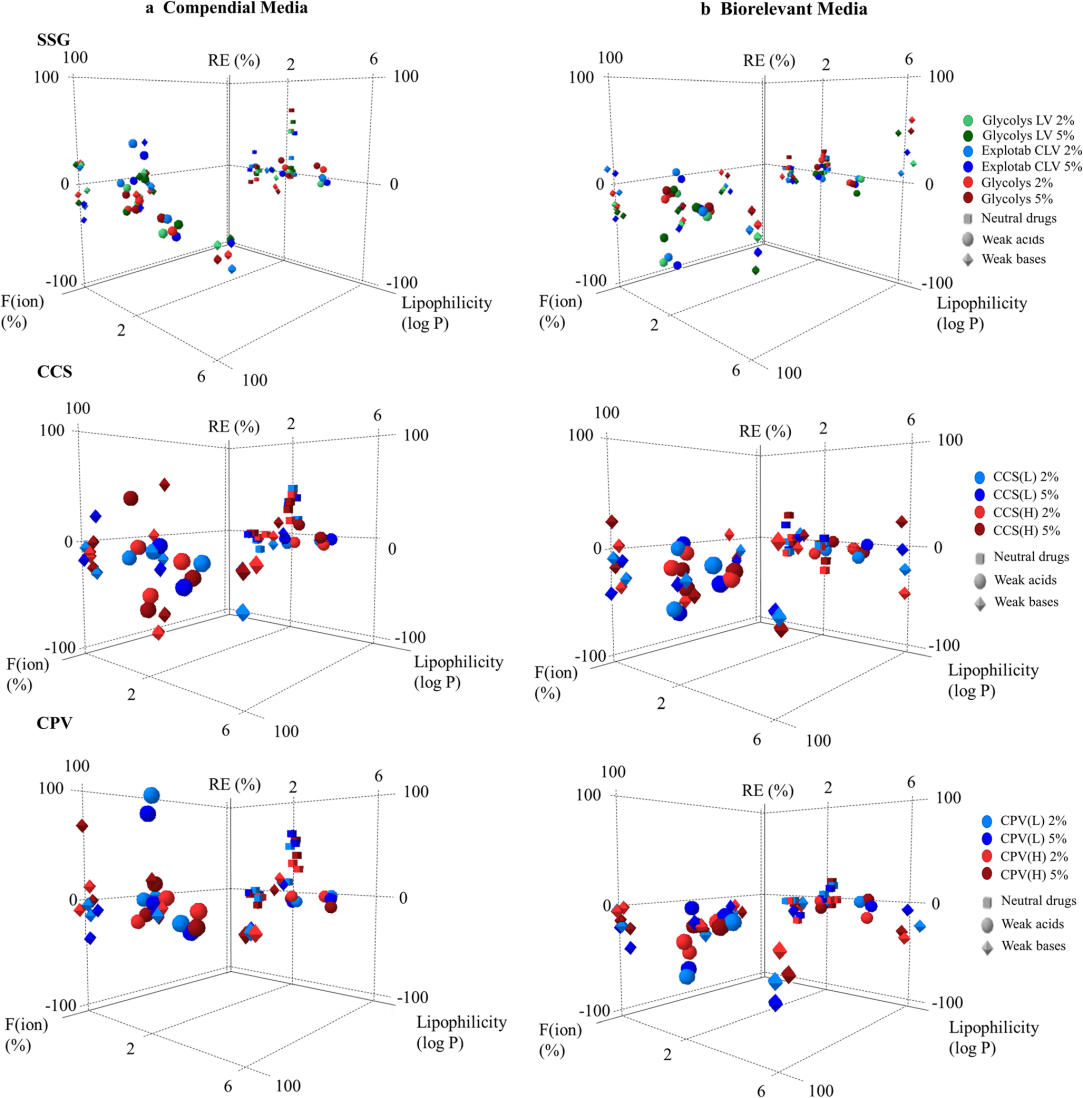
Relative effects (%) of the studied SSG (Glycolys LV (green colour), Explotab CLV (blue colour), Glycolys (red colour)), CCS (CCS(L) (blue colour), CCS(H) (red colour)) and CPV (CPV(L) (blue colour), CPV(H) (red colour)) brands on drug solubility at 24 h as a function of drug ionisation (%) and drug lipophilicity (log *P*) in **a** compendial and **b** biorelevant media. Light and dark colours correspond to low and high excipient level, respectively

**Fig. 6:**
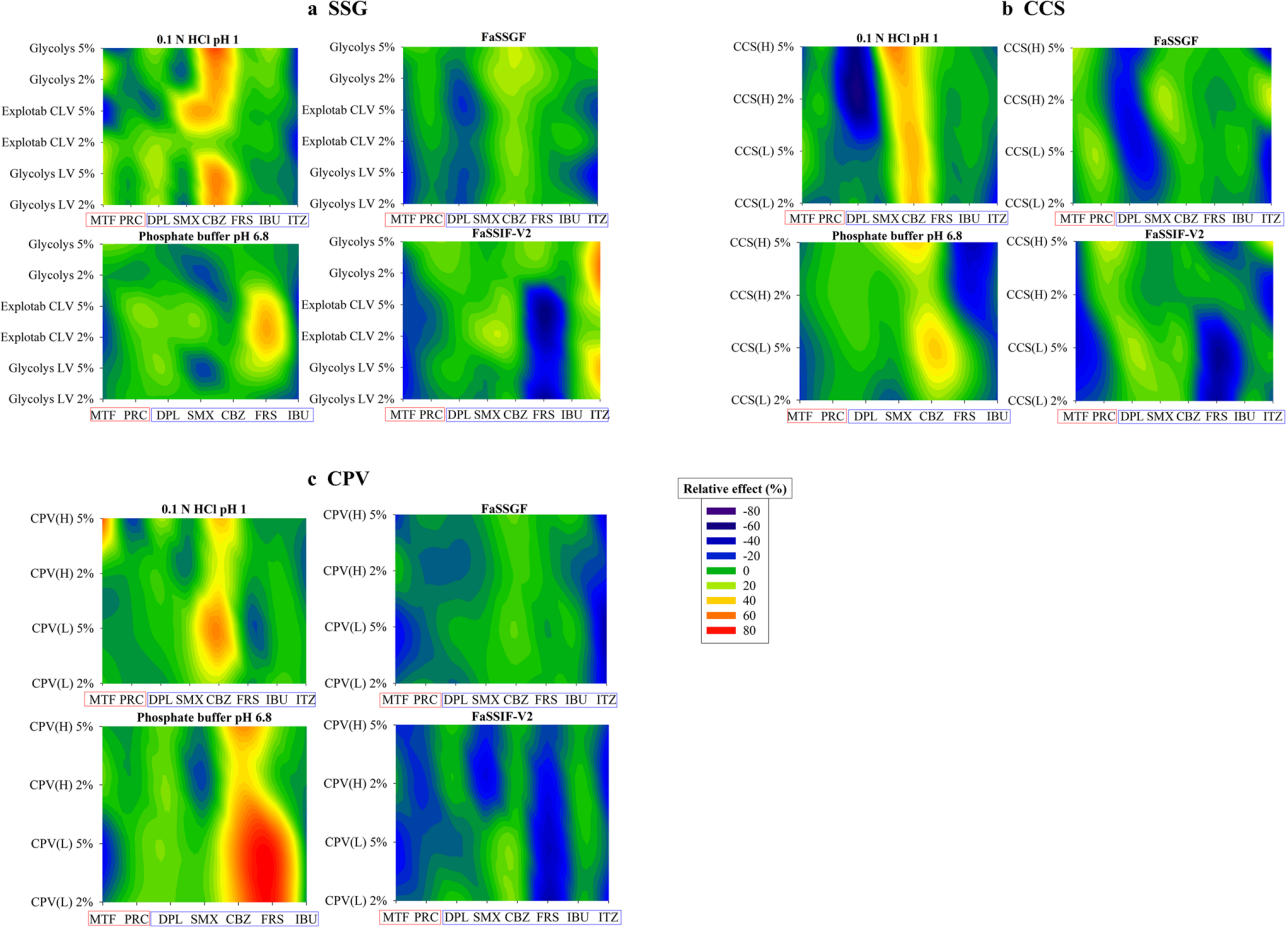
Classification gradient maps of the relative excipient effects of the **a** SSG, **b** CCS and **c** CPV brands on the solubility of highly and poorly soluble compounds at 24 h. *Y*-axes are set in an increasing viscosity and level order for SSG and increasing particle size and level order for CCS and CPV. The *x*-axes are set in a decreasing drug aqueous solubility order (red colours for highly soluble and blue colours for poorly soluble drugs)

The effects of the studied superdisintegrants on drug solubility at 24 h as a function of drug ionisation and drug lipophilicity in compendial and biorelevant media are presented in Fig. 5. The reduction in drug apparent solubility by superdisintegrant presence is more pronounced in media (compendial or biorelevant) where drugs are highly ionised (excluding the cases of increased drug solubility attributed to the change in the pH of the medium), potentially due to the presence of a high number of excipient particles on the powder surface which limits drug dissolution and/or drug solubilisation (48). For the ionic superdisintegrants (SSG, CCS), interactions between ionised drugs and the excipient polymeric chains (17,59) may also have contributed to the observed reduction in drug apparent solubility. A trend between the impact of superdisintegrants on drug apparent solubility and drug lipophilicity was not observed, apart from the case of SSG in biorelevant media, where an increase in drug solubility at 24 h was observed with increasing drug lipophilicity (when drugs are in the low ionisation state). The classification gradient maps depicting the effects of the studied superdisintegrants on drug solubility at 24 h as a function of drug aqueous solubility in compendial and biorelevant media is presented in Fig. 6. A clear trend between the reduction in drug solubility by excipient presence and drug aqueous solubility cannot be observed.

### Multivariate Data Analysis

**Fig. 7:**
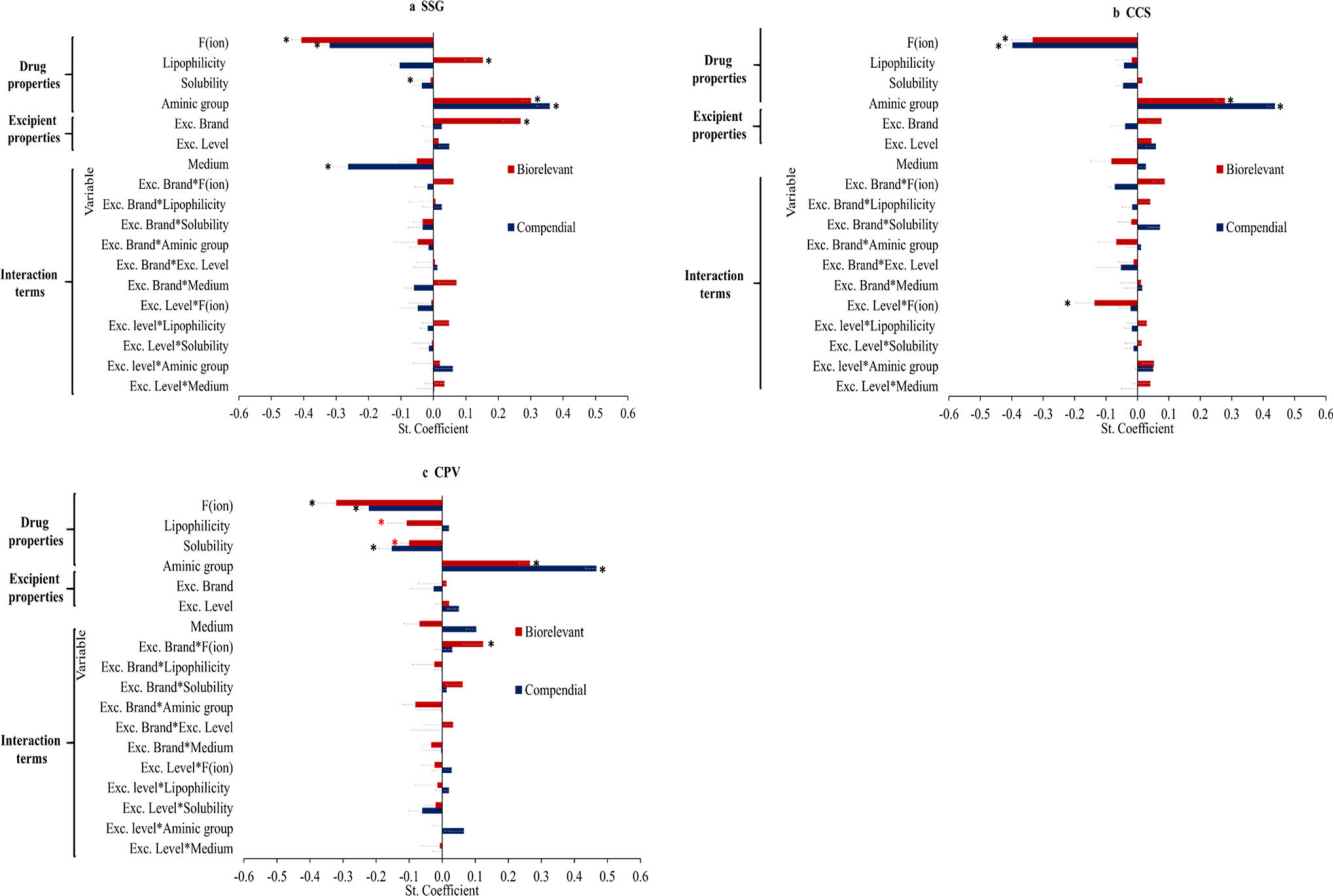
Standardised coefficients of the studied variables (and interaction terms) in compendial (blue colour) and biorelevant (red colour) media for **a** SSG, **b** CCS and **c** CPV. * denotes coefficients of VIP > 1 (mean, − SE)

For SSG, the two models showed an average fit (compendial media—1 principal component, *Q*^2^ = 0.3, *R*^2^ = 0.4; biorelevant media—2 principal components, *Q*^2^ = 0.4, *R*^2^ = 0.5) (Fig. 7a). The statistical model reveals that the impact of SSG on drug apparent solubility depends on drug physicochemical properties. Amine group (compendial media—positive effect, VIP = 2.7; biorelevant media—positive effect, VIP = 2.3) was a significant variable in both sets of media indicating that a significant increase in drug solubility at 24 h is anticipated in SSG presence for drugs containing a neutral amine due to potential drug–SSG interaction which improves drug solubilisation (54). Drug ionisation (compendial media—negative effect, VIP = 2.4; biorelevant media—negative effect, VIP = 2.5) was an influential variable in both models indicating that significant reduction in drug apparent solubility in SSG presence is expected for highly ionised drugs due to the saturation of powder surface with excipient particles (48) or drug–SSG interactions (17) which delay drug dissolution and/or drug solubilisation. In biorelevant media, drug lipophilicity (positive effect, VIP = 1.4) and drug solubility (negative effect, VIP = 1.0) were significant variables in the model. These variables indicate that pronounced increase in the apparent solubility of poorly soluble/lipophilic drugs can be observed in presence of SSG as a result of enhanced drug solubilisation. The negative effect of drug solubility can also indicate a reduction in drug solubility at 24 h for highly soluble drugs due to the saturation of powder surface with excipient particles (48) (as for highly soluble drugs, drug molecules can dissolve faster in the medium especially in the presence of solubilising components (45)). The impact of excipient properties on drug apparent solubility was found critical only in biorelevant media as demonstrated by the significance of the term exc. Brand (positive effect, VIP = 1.4) in the model. This term reveals that the increase in drug apparent solubility will be more pronounced in presence of high-viscosity SSG brands as potentially high-viscosity excipients have a better ability in delaying particle agglomeration and improve drug solubilisation, influencing the drug apparent rather than the true equilibrium drug solubility. The impact of the ionisation pattern of SSG on drug dissolution due to its higher swelling in basic media is revealed by the significant negative effect of the variable medium in the compendial model (VIP = 1.9); this effect was not observed in the biorelevant model probably due to the presence of other components in the media (8).

For CCS, average fittings (compendial media—1 principal component, *Q*^2^ = 0.5, *R*^2^ = 0.6; biorelevant media—1 principal component, *Q*^2^ = 0.2, *R*^2^ = 0.3) were obtained (Fig. 7b). Amine group (compendial media—positive effect, VIP = 3.0; biorelevant media—positive effect, VIP = 2.4) and drug ionisation (compendial media—negative effect, VIP = 2.7; biorelevant media—negative effect, VIP = 2.8) were significant factors in both sets of media. The variable amine group indicates that a significant increase in the apparent solubility of drugs containing a neutral amine group is expected as a result of the enhanced drug solubilisation by CCS presence due to a potential drug–CCS interaction which improves drug solubilisation (54). The negative effect of drug ionisation reveals that pronounced reduction in the 24 h solubility of highly ionised drugs will be anticipated in presence of CCS due to the saturation of the powder surface by excipient particles (48) or drug–CCS interactions (17) which limit drug dissolution and/or drug solubilisation. Excipient properties can be critical factors for the impact of CCS on drug apparent solubility in biorelevant media, as demonstrated by the significance of the variable excipient level × drug ionisation (negative effect, VIP = 1.1) in the model. As the presence of solubilising components improves powder wettability and drug solubilisation (45), the high number of excipient particles on top of the powder surface or the extensive excipient swelling when increasing CCS level will result in higher reduction in the apparent solubility of highly ionised drugs.

For CPV, average fits were observed (compendial media—1 principal component, *Q*^2^ = 0.4, *R*^2^ = 0.5; biorelevant media—1 principal component, *Q*^2^ = 0.2, *R*^2^ = 0.3) (Fig. 7c). Drug physicochemical properties were critical parameters for the impact of CPV on drug solubility. Amine group (compendial media—positive effect, VIP = 3.5; biorelevant media—positive effect, VIP = 2.3) was a significant variable in both models indicating that CPV is able in inhibiting drug agglomeration (54). Drug ionisation (compendial media—negative effect, VIP = 1.6; biorelevant media—negative effect, VIP = 2.8) and drug solubility (compendial media—negative effect, VIP = 1.1; biorelevant media—negative effect, VIP = 0.9) were significant variables in both models. Both variables indicate that a significant reduction in drug apparent solubility is anticipated in presence of CPV for highly ionised or highly soluble drugs, potentially due to the saturation of the powder surface with excipient particles (48). In biorelevant media, drug lipophilicity (negative effect, VIP = 0.9) was a significant factor in the model indicating significant reduction in the apparent solubility of highly lipophilic drugs in presence of CPV. The enhanced drug solubilisation of lipophilic molecules by the presence of bile salts in biorelevant conditions (45) may result in saturation of the powder surface with excipient particles which further limit drug dissolution and/or drug solubilisation. Hydrophobic interactions between lipophilic drugs and CPV (17) could also have contributed to the delay in drug dissolution in excipient presence. Finally, the interaction exc. Brand × drug ionisation (positive effect, VIP = 1.1) was a significant variable in the biorelevant model, but further investigations are needed to explain the nature of this term.

### Roadmap of Superdisintegrants’ Effects on Drug Apparent Solubility

**Fig. 8:**
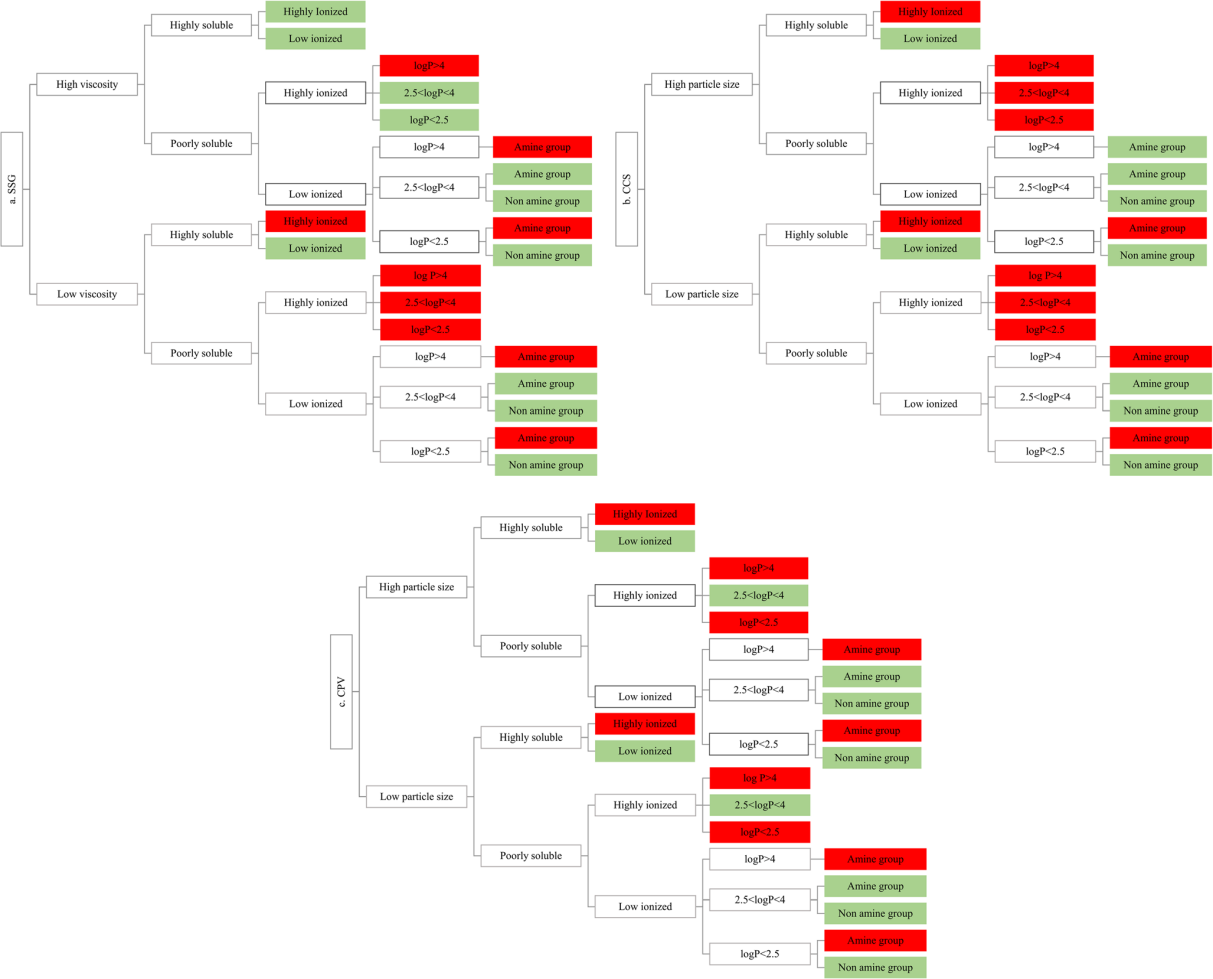
Road map of the effects of the studied **a** SSG, **b** CCS and **c** CPV brands on drug solubility. Red boxes and green boxes indicate significant and insignificant changes in drug solubility by excipient presence, respectively

The roadmaps categorising excipient REs on drug apparent solubility according to excipient (SSG, CCS, CPV) and drug properties are presented in Fig. 8 (cases where increased drug solubility was caused by a potential shift in the pH of the medium were not considered).

The impact of the studied superdisintegrants on drug solubility relates to drug physicochemical properties. Presence of low-viscosity SSG brands needs to be further studied as it may be critical in oral drug performance for highly ionised drugs, irrespective of drug lipophilicity or drug aqueous solubility, as cases of pronounced reduction in drug solubility at 24 h in presence of low-viscosity SSG brands were observed (Glycolys LV, Explotab CLV). High-viscosity SSG (Glycolys) brands will be challenging for the oral performance of poorly soluble/highly ionised drugs with log *P* > 4. For poorly soluble/low ionised drugs, presence of SSG is not expected to affect drug apparent solubility, apart from drugs containing a neutral amine group and for which SSG presence may result in significant increase in drug apparent solubility (Fig. 8a).

The criticality of CCS for oral product performance relates to drug ionisation as significant changes (decrease) in drug apparent solubility in CCS presence are expected for highly ionised drugs, irrespective of drug aqueous solubility (highly or poorly soluble drugs). Moreover, presence of CCS will be critical for the solubility of poorly soluble/low ionised drugs (log *P* < 2.5) containing a neutral amine group, as significant increase in the 24-h drug solubility was observed (Fig. 8b). Presence of low particle size CCS brands may be challenging for the solubility of poorly soluble/low ionised drug with log *P* > 4; however, its impact on drug solubility depends on excipient level (Fig. 3).

The impact of CPV on drug solubility depends on drug ionisation and drug lipophilicity, as significant changes (reduction) were observed in the apparent solubility of highly ionised/highly soluble or highly ionised/poorly soluble drugs with log *P* < 2.5, irrespective of excipient brand used. Presence of CPV can also be critical for the solubility of highly lipophilic drugs (log *P* > 4), irrespective of drug ionisation state (highly or low ionised), as significant reduction in drug solubility at 24 h was observed by all the studied CPV brands. Finally, presence of CPV may present challenges in the oral drug performance of poorly soluble/low ionised drugs with log *P* < 2.5 as significant increase in drug apparent solubility was observed (Fig. 8c).

The construction of roadmaps identified the cases where presence of superdisintegrants in solid oral dosage forms needs to be examined in order to better understand the impact of this excipient on oral drug performance. Compared to lubricants (60) and binders (61), superdisintegrants can be considered as excipients of low criticality for formulation performance, when considering the impact of these excipients on drug solubility alone (the impact of superdisintegrant variability on tablet disintegration could still be of high risk for oral drug bioavailability).

## CONCLUSIONS

Superdisintegrant variability and interchangeability present challenges in pharmaceutical development, as the varying excipient physicochemical properties can affect final product quality. Identification of the critical excipient attributes affecting product performance is recommended for the successful control of excipient variability according to the QbD approach. Presence of superdisintegrants (SSG, CCS, CPV) in immediate-release formulations is beneficial for promoting fast tablet disintegration and drug dissolution, but there is a lack of knowledge on the impact of their properties on oral drug performance. In this work, the biopharmaceutical implications of superdisintegrant variability (viscosity type for SSG, particle size distribution for CCS and CPV) on drug apparent solubility were investigated. A data set for the initial risk assessment of the impact of superdisintegrants on oral drug performance was generated and revealed that for the majority of cases, presence of superdisintegrants or superdisintegrant variability did not significantly affect drug apparent solubility. The significant changes in drug solubility at 24 h related to drug physicochemical properties. Reduction in drug apparent solubility was observed for highly ionised drugs and attributed to the adsorption of superdisintegrants around drug particles. Presence of superdisintegrants increased the apparent solubility of poorly soluble drugs containing a neutral aminic group related most probably to drug–excipient interactions or inhibition of drug agglomeration. A clear trend between the excipient effects on drug apparent solubility and drug lipophilicity was not observed. The use of multivariate data analysis and the design of roadmaps allowed the identification of the biopharmaceutical factors affecting the impact of superdisintegrants on drug apparent solubility. Although a limited amount of compounds was included in this study and molecular descriptors were not taken into account for the assessment of the excipient effects on drug solubility, the absence of significant effects on drug solubility in the presence of the studied excipients reveals that, compared to other excipient types (lubricants, binders), superdisintegrants can be considered as of low biopharmaceutical criticality for presenting implications on oral drug absorption.

## Electronic supplementary material

ESM 1 (PDF 1189 kb)
