# Supplementary material for: Biopharmaceutical Understanding of Excipient Variability on Drug Apparent Solubility Based on Drug Physicochemical Properties. Case Study: Superdisintegrants
Source: AAPS J. 2020 Feb 11;22(2):46. doi: 10.1208/s12248-019-0406-y (PMC7012964; doi:10.1208/s12248-019-0406-y)
Supplement: Supplementary file 1 — (PDF 1189 kb) [file 12248_2019_406_MOESM1_ESM.pdf]

Supplementary Table I: HPLC methods used for drug quantification

	Column	Mobile Phase	Flow Rate (mL/ min)	Temp (°C)	Inj. Vol. (µL)	Detection wavelength (nm)	R_t (min)	Concentration of stock solutions (mg/mL)	Calibration range in acidic media (µg/mL)	Calibration range in basic media (µg/mL)	Reference
**MTF**	Inertsil Phenyl (Metachem) 250x3mm - 5µm	MeOH/ Phosphate buffer pH 7 (70:30)	1	20	20	236	8	2	10 - 200	10 - 200	(1)
**PRC**	Spherisorb (Waters) C18 250x4.6 mm - 5µm	MeOH/ Water (20:80)	1	20	20	257	6	2	10 - 200	10 - 200	(2)
**SMX**	Polaris (Metachem) C18 250x4.6 mm – 5µm	MeOH/ Phosphate buffer pH 6.8 (20:80)	1	25	20	257	7	1	10 - 200	50 – 500	(3)
**FRS**	Spherisorb (Waters) C18 250x4.6 mm - 5µm	MeOH/ water with 0.1% formic acid (50:50)	1	25	50	232	4	1	2 – 20	10 - 200	(4)

**CBZ**	Spherisorb (Waters) C18 250x4.6 mm - 5µm	MeOH/ Water (60:40)	1	25	100	285	4	1	10 – 150	10 - 150	(5)
**DPL**	XBridge Shield C18 150x4.6 mm – 3.5µm	ACN/water with 0.1% TFA (30:70)	1	25	50	284	6	1	10 -200	Compendial 1 - 5 Biorelevant 2 - 10	(6)
**IBU**	Eclipse XDB-C18(Agilent) 250x4.6 mm – 5µm	MeOH/ water with 0.2% acetic acid (65:35)	1	25	100	233	6	1	5 – 40	10 - 200	(7)
**ITZ***	XBridge Shield C18 150x4.6 mm – 3.5µm	ACN/phosphate buffer pH 3 (60:40)	1	20	100	Emission: 252 Excitation: 360	8	0.1	Compendial 0.5 – 5 Biorelevant 0.1 - 10	0.015 – 0.06	(8)

*Quantification was made using HPLC-Fluorescence. MTF = metformin, PRC = paracetamol, SMX = sulfamethoxazole, FRS = furosemide, CBZ = carbamazepine, DPL = diyridamole, IBU = ibuprofen, ITZ = itraconazole

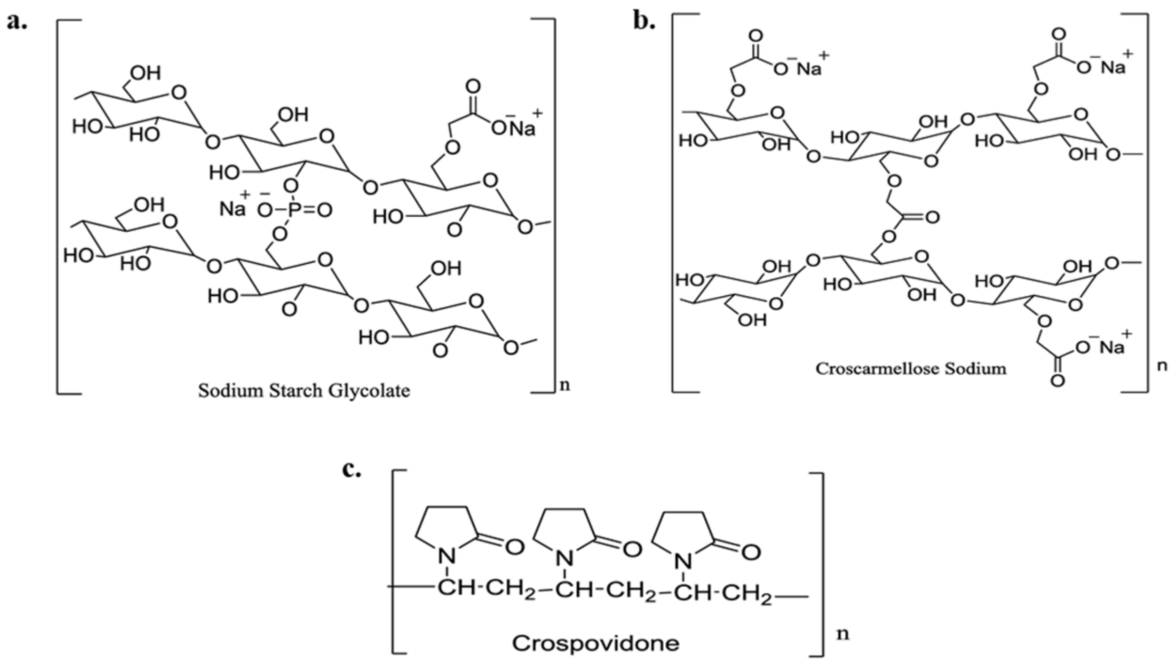

Supplementary Figure 1: Chemical structure of a. Sodium Starch Glycolate, b. Croscarmellose Sodium and c. Crospovidone (ChemDraw Professional 15).

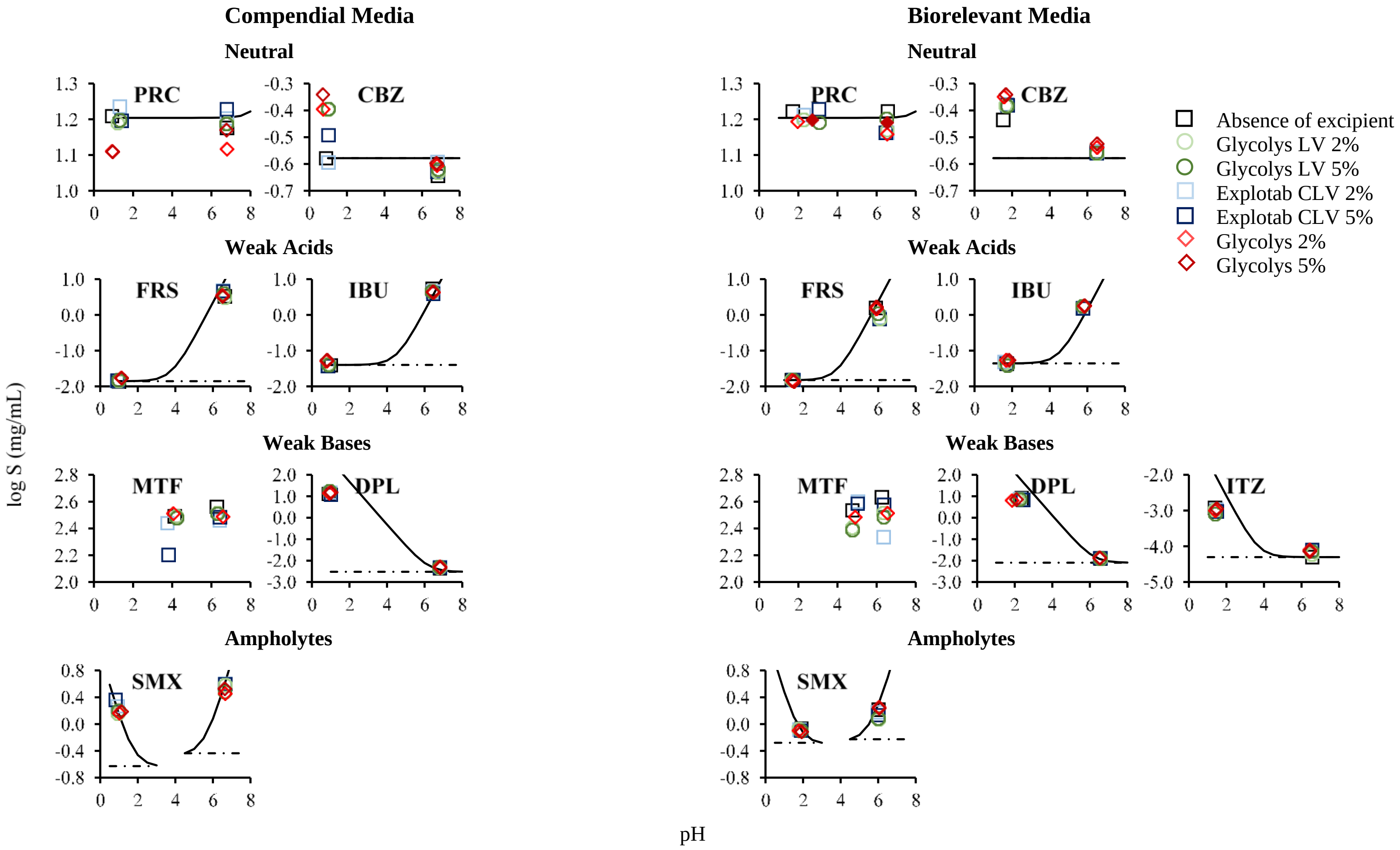

Supplementary Figure 2: Theoretical pH-solubility profiles of the studied drugs in compendial and biorelevant media and experimental drug solubility values in absence (black colour) and presence of excipients (i. Glycolys LV (green colour), ii. Explotab CLV (blue colour), iii. Glycolys (red colour)). Dashed lines indicate drug intrinsic solubility.

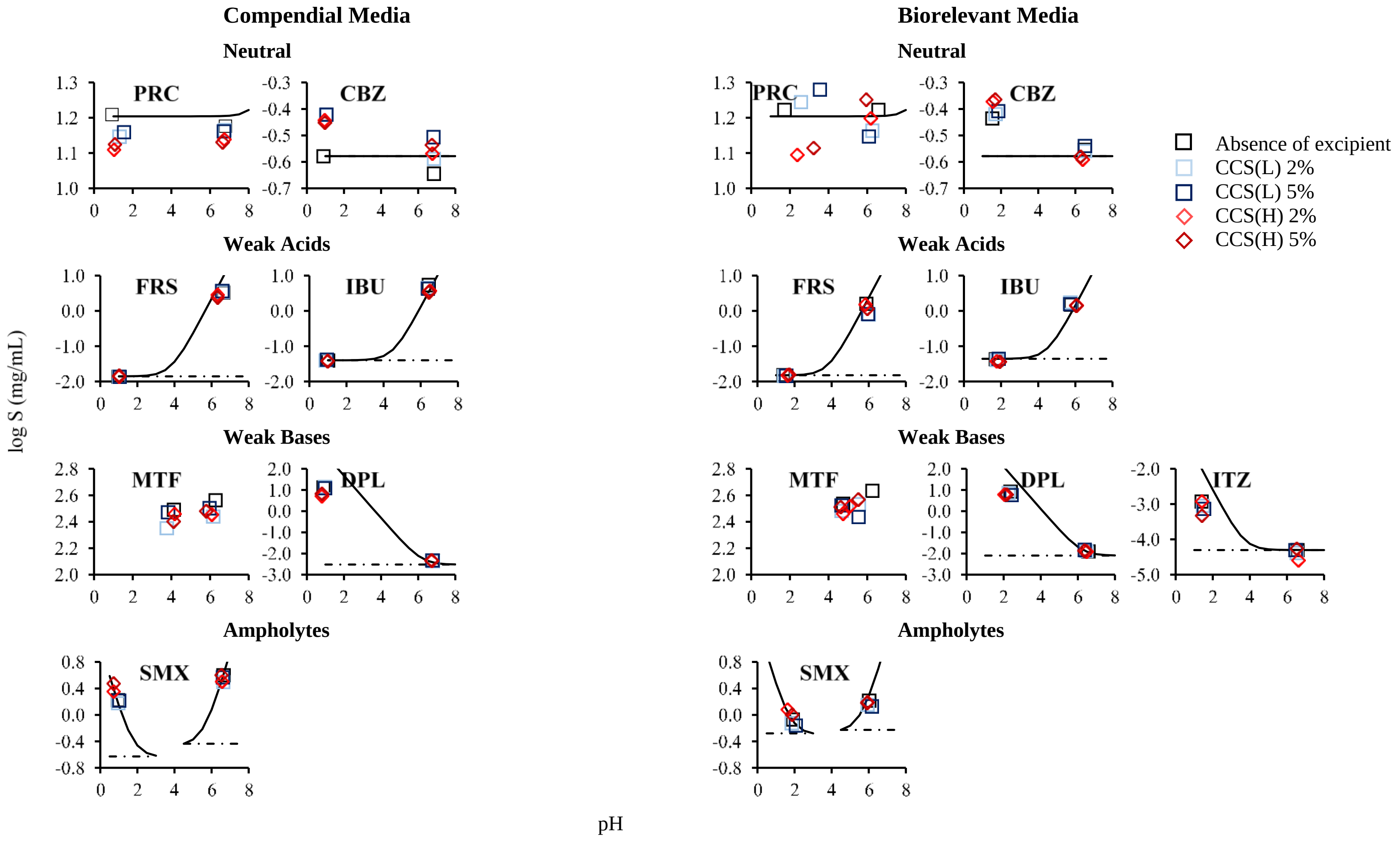

Supplementary Figure 3: Theoretical pH-solubility profiles of the studied drugs in compendial and biorelevant media and experimental drug solubility values in absence (black colour) and presence of excipients (i. CCS(L) (blue colour), ii. CCS(H) (red colour)). Dashed lines indicate drug intrinsic solubility.

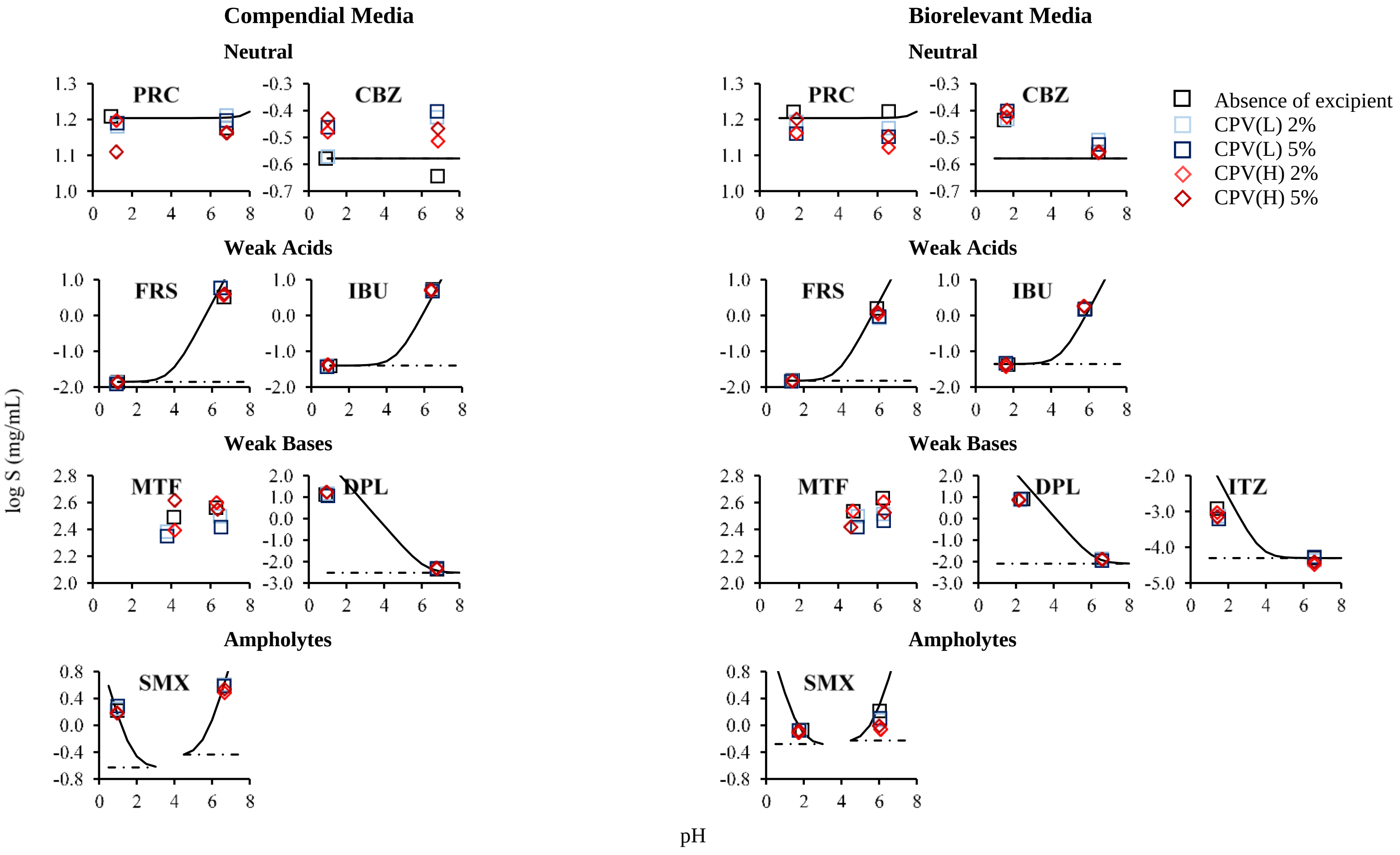

Supplementary Figure 4: Theoretical pH-solubility profiles of the studied drugs in compendial and biorelevant media and experimental drug solubility values in absence (black colour) and presence of excipients (i. CPV(L) (blue colour), ii. CPV(H) (red colour)). Dashed lines indicate drug intrinsic solubility.

References

1. Porta V, Schramm SG, Kano EK, Koono EE, Armando YP, Fukuda K, et al. HPLC-UV determination of metformin in human plasma for application in pharmacokinetics and bioequivalence studies. JPharmBiomedAnal. 2008;46(1):143-7.

2. Gao N, Qi B, Liu FJ, Fang Y, Zhou J, Jia LJ, et al. Inhibition of baicalin on metabolism of phenacetin, a probe of CYP1A2, in human liver microsomes and in rats. PLoS One. 2014;9(2):e89752.

3. Vree TB, Hekster YA, Baars AM, Damsma JE, Kleijin EV. Determination of trimethoprim and sulfamethoxazole (co-trimoxazole) in body fluids of man by means of high-performance liquid chromatography. Journal of chromatography. 1978;146(1):103-12.

4. Sora DI, Udrescu S, Albu F, David V, Medvedovici A. Analytical issues in HPLC/MS/MS simultaneous assay of furosemide, spironolactone and canrenone in human plasma samples. JPharmBiomedAnal. 2010;52(5):734-40.

5. Vertzoni MV, Reppas C, Archontaki HA. Sensitive and simple liquid chromatographic method with ultraviolet detection for the determination of nifedipine in canine plasma. Analytica Chimica Acta. 2006;573-574(Supplement C):298-304.

6. Soderlind E, Karlsson E, Carlsson A, Kong R, Lenz A, Lindborg S, et al. Simulating fasted human intestinal fluids: understanding the roles of lecithin and bile acids. MolPharm. 2010;7(5):1498-507.

7. Tan A, Eskandar NG, Rao S, Prestidge CA. First in man bioavailability and tolerability studies of a silica-lipid hybrid (Lipoceramic) formulation: a Phase I study with ibuprofen. DrugDelivTranslRes. 2014;4(3):212-21.

8. Ghazal HS, Dyas AM, Ford JL, Hutcheon GA. In vitro evaluation of the dissolution behaviour of itraconazole in bio-relevant media. IntJPharm. 2009;366(1):117-23.
